# Heterologous Exchanges of Glycoprotein and Non-Virion Protein in Novirhabdoviruses: Assessment of Virulence in Yellow Perch (*Perca flavescens*) and Rainbow Trout (*Oncorhynchus mykiss*)

**DOI:** 10.3390/v16040652

**Published:** 2024-04-22

**Authors:** Vikram N. Vakharia, Arun Ammayappan, Shamila Yusuff, Tarin M. Tesfaye, Gael Kurath

**Affiliations:** 1 Institute of Marine & Environmental Technology, University of Maryland Baltimore County, Baltimore, MD 21202, USA; arunvet2005@gmail.com (A.A.); umub12@gmail.com (S.Y.); 2 Former U.S. Geological Survey, Seattle, WA 98115, USA; tarin.marie@gmail.com; 3 U.S. Geological Survey, Western Fisheries Research Center, Seattle, WA 98115, USA

**Keywords:** IHNV, VHSV, viral pathogenesis, rhabdovirus, glycoprotein, non-virion protein

## Abstract

Infectious hematopoietic necrosis virus (IHNV) and viral hemorrhagic septicemia virus (VHSV) are rhabdoviruses in two different species belonging to the *Novirhabdovirus* genus. IHNV has a narrow host range restricted to trout and salmon species, and viruses in the M genogroup of IHNV have high virulence in rainbow trout (*Oncorhynchus mykiss*). In contrast, the VHSV genotype IVb that invaded the Great Lakes in the United States has a broad host range, with high virulence in yellow perch (*Perca flavescens*), but not in rainbow trout. By using reverse-genetic systems of IHNV-M and VHSV-IVb strains, we generated six IHNV:VHSV chimeric viruses in which the glycoprotein (G), non-virion-protein (NV), or both G and NV genes of IHNV-M were replaced with the analogous genes from VHSV-IVb, and vice versa. These chimeric viruses were used to challenge groups of rainbow trout and yellow perch. The parental recombinants rIHNV-M and rVHSV-IVb were highly virulent in rainbow trout and yellow perch, respectively. Parental rIHNV-M was avirulent in yellow perch, and chimeric rIHNV carrying G, NV, or G and NV genes from VHSV-IVb remained low in virulence in yellow perch. Similarly, the parental rVHSV-IVb exhibited low virulence in rainbow trout, and chimeric rVHSV with substituted G, NV, or G and NV genes from IHNV-M remained avirulent in rainbow trout. Thus, the G and NV genes of either virus were not sufficient to confer high host-specific virulence when exchanged into a heterologous species genome. Some exchanges of G and/or NV genes caused a loss of host-specific virulence, providing insights into possible roles in viral virulence or fitness, and interactions between viral proteins.

## 1. Introduction

Infectious hematopoietic necrosis virus (IHNV) and viral hemorrhagic septicemia virus (VHSV) cause severe disease and mortality in many freshwater and marine fish species in North America, Europe, and North Asia [1,2,3]. These viruses produce severe hemorrhages in the skin, muscles, eyes, kidneys, and liver, with mortality rates as high as 90% [4,5]. Both IHNV and VHSV are enveloped, non-segmented, negative-sense RNA viruses in the *Novirhabdovirus* genus of the *Rhabdoviridae* family [6,7]. Their genomes are composed of approximately 11 kb of single-stranded RNA, which contains six genes that are located along the genome in the 3′ to 5′ order: 3′-N-P-M-G-NV-L-5′, nucleocapsid protein (N), polymerase-associated phosphoprotein (P), matrix protein (M), surface glycoprotein (G), a unique non-virion protein (NV), and a large protein (L), which is a viral polymerase [8,9]. The viruses replicate entirely in the cytoplasm by means of a combination of virus-encoded and host-derived factors. The functions of the five canonical rhabdovirus proteins have been defined largely based on studies of the mammalian rabies virus (RABV) and vesicular stomatitis virus (VSV), and their roles are considered universal for rhabdoviruses [6,10]. N is the major internal structural protein, which encapsidates the RNA genome. L protein is a polymerase essential for viral RNA transcription and genome replication activity [11]. P protein is a cofactor responsible for binding L protein to the N protein–RNA template, and it has a chaperone role for N protein. Together, N, P, and L proteins form the functional viral ribonucleoprotein (RNP) complex [11]. The M protein condenses the RNP into a tightly coiled RNP-M protein complex, which gives the virion a bullet-like shape. G glycoprotein forms trimer surface spikes that protrude through the cell-derived envelope and interact with host cell receptors to facilitate cell entry [12]. An additional gene located between the G and L cistrons is unique to novirhabdoviruses and codes for a non-virion NV protein. This NV protein is required for efficient replication and pathogenicity in fish [13,14,15,16], and it suppresses apoptosis and modulates the innate immune responses [17,18,19].

Viral hemorrhagic septicemia (VHS) disease, first described in freshwater-reared rainbow trout in Europe in 1938, has continued to plague the European trout farming industry since the 1950s [1,3,5]. In the late 1980s, VHSV was isolated for the first time in western North America from adult Coho salmon (*Oncorhynchus kisutch*) and Pacific cod (*Gadus macrocephalus*), but the virus was not virulent for rainbow trout (*O. mykiss*) [20]. In 2005, a new genotype of VHSV, designated IVb, emerged in the Great Lakes region of the United States and Canada, which caused massive mortality in 28 diverse freshwater fish species because of the broad host range of VHSV [21,22,23,24,25]. Based on phylogenetic studies, global VHSV isolates are geographically distributed into four genotypes, I-IV, which are further classified into sub-lineages and strains [26,27,28,29,30] that sometimes differ in host-specific virulence. Viruses in the VHSV subgroup IVb from the Great Lakes caused numerous large fish kills in yellow perch (*Perca flavescens*) and show high perch virulence in experimental infection studies, but they have low virulence in rainbow trout [25,31,32].

IHNV is an important pathogen causing disease impacts in salmon and trout in the United States since it was first reported in the 1950s [2,4]. IHNV causes an acute disease with high mortality similar to VHSV, but it differs in having a narrow host range limited to species in the family *Salmonidae*, which includes Pacific salmon and trout. IHNV epidemics were first reported in salmon hatcheries rearing sockeye (*O. nerka*) and Chinook (*O. tshawytscha*) salmon, but the virus adapted to rainbow trout and emerged in trout farms of southern Idaho in the late 1970s [33]. IHNV has been inadvertently introduced to Europe and Asia, where it causes increasing losses in cultured rainbow trout [34,35]. Phylogenetic analyses based on complete or partial G gene sequences of IHNV isolates from worldwide sources have defined five major genogroups: U (upper), M (middle), L (lower), E (Europe), and J (Japanese/Asian) [36]. Genetic analyses and evolutionary dynamics reveal that IHNV is evolving relatively rapidly in rainbow trout [37,38]. In North America, IHNV in the M genogroup has high virulence in rainbow trout, but low virulence in yellow perch [39,40].

Phylogenetic analyses have shown that both VHSV and IHNV have adapted by host jumps into cultured rainbow trout in the past, resulting in the divergence of new phylogenetic lineages in Europe and North America, respectively [26,27,28,33,36]. Given the presence of VHSV-IVb in the Great Lakes, it has the potential to jump into rainbow trout, and possibly other aquaculture species [41]. However, the genetic changes associated with viral host jumps and host-specific virulence of fish rhabdoviruses are not well understood. Previous studies have shown that the VHSV-IVb strain MI03 is very virulent in yellow perch, but not in rainbow trout [31,32], whereas IHNV-M strain 220-90 is very virulent in rainbow trout, but not in yellow perch [39,40].

In this study, to explore the viral genetic basis of virulence and host specificity of VHSV-IVb and IHNV-M, we utilized the infectious clones developed previously for VHSV strain MI03 and IHNV strain 220-90 [15,42]. We generated six IHNV:VHSV chimeric viruses in which the glycoprotein (G), non-virion protein (NV), or both G and NV genes of IHNV-M were replaced with the analogous genes from the VHSV-IVb, and vice versa. Viable viruses were recovered in all cases. Here, we determine the virulence of these chimeric viruses in both rainbow trout and yellow perch to assess the role of the G and NV genes in novirhabdovirus host-specific virulence.

## 2. Materials and Methods

**Viruses and cells:** The North American MI03 strain of VHSV (species *Salmonid novirhabdovirus*) genotype IVb was obtained from Dr. M. Faisal at Michigan State University. This virus was isolated from muskellunge, *Esox masquinongy* (Mitchill), caught from the NW portion of Lake St Clair, Michigan, USA, in 2003 [21]. The clinical signs of disease observed in dead and moribund fish were very pale gills, dermal petechial hemorrhages along the flanks, severe nuchal hemorrhages, intramuscular hemorrhages at the fin–muscle junction, and focal hemorrhaging on the caudal peduncle. In 2009, we cloned the genome of this virus and submitted the complete nucleotide sequence of the VHSV-MI03 strain to the GenBank database (accession no. GQ385941). The virulent 220-90 strain of IHNV (species Novirhabdovirus piscine) genogroup M was provided by Dr. Scott LaPatra of Clear Springs Foods Inc., Buhl, Idaho, USA. The virus was isolated from juvenile rainbow trout during routine examinations of hatchery-reared fish, conducted from 1990 to 1992 in the Hagerman Valley, ID, USA [43]. The affected fish showed cumulative mortality of 90% with the gross pathological signs including exophthalmia and hemorrhage of the eye, skin darkening, abdominal distension, ulceration of the snout, and visceral pallor and yellowish fluid in the intestine. In 2010, we cloned the genome of this virus (which is still used as a challenge virus to date) and deposited the complete nucleotide sequence of the IHNV-220-90 strain into GenBank (accession no. GQ413939). These parental virus strains and all recombinant VHSVs and IHNVs generated here were propagated in the *Epithelioma papulosum cyprini* (EPC) cyprinid fish cell line at 14 °C, as previously described [15,42]. Briefly, EPC cells were propagated in minimum essential medium (MEM) supplemented with 10% fetal bovine serum and 2 mM L-glutamine (ATCC, Manassas, VA, USA). For routine cell propagation, the EPC cells were incubated at 28 °C. To propagate the virus, the cells were infected and incubated at 14 °C until cytopathic effects (CPE) were complete. The supernatant was collected 5 days post-infection, clarified, and stored at −80 °C for further processing.

**Figure 1:**
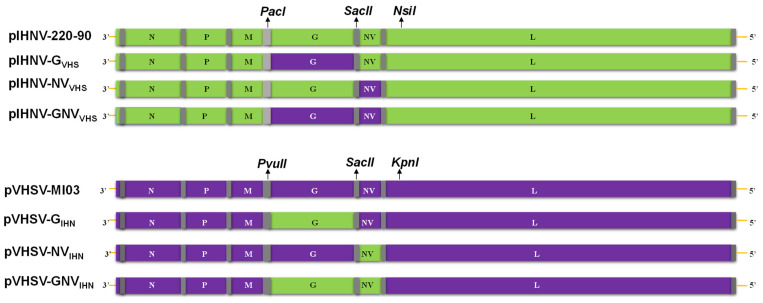
Schematic presentation of full-length parental and chimeric cDNA clones of the IHNV-220-90 series and the VHSV-MI03 series. Each full-length infectious clone of an IHNV, VHSV, or chimeric genome contains the coding regions of N, P, M, G, NV, and L genes, which are separated by the flanking regulatory untranslated and intergenic sequences (vertical bars). Selected restriction sites, important for the construction of chimeric cDNA clones, are as indicated. These unique sites are present in the intergenic regions of the clones, which does not affect the coding regions of viral proteins. All of these constructs contain a cytomegalovirus promoter at the 3′-end. The upper four genomes illustrate the rIHNV-220-90 series based on the parental infectious clone of IHNV strain 220-90, with chimeric cDNA clones derived by substitution of G and/or NV genes with genes from VHSV strain MI03. The lower four genomes illustrate the rVHSV-MI03 series based on the parental infectious clone of VHSV strain MI03, with chimeric cDNA clones derived by substitution of G and/or NV genes with genes from IHNV strain 220-90.

**Construction of chimeric cDNA clones of VHSV and IHNV:** The construction of infectious clones of the VHSV-MI03 and IHNV-220-90 strains has been described previously [15,42]. The full-length plasmid clones of the VHSV-MI03 and IHNV-220-90 strains were designated as pVHSV-MI03 and pIHNV-220-90, respectively (Figure 1). These clones were used as backbones to introduce the desired substitution(s) of the G, NV, or G and NV protein genes of reciprocal VHSV or IHNV strains. To construct chimeric cDNA clones of IHNV and VHSV, specific primer pairs were used to amplify DNA fragments, which contained the ORF sequences of G, NV, or G and NV, whilst maintaining the intergenic sequences of the individual pVHSV-MI03 or pIHNV-220-90 backbone. These DNA fragments were cloned between the unique restriction sites (PacI/PvuII, SacII, NsiI/KpnI) present in the noncoding regions of individual pIHNV-220-90 or pVHSV-MI03 plasmids to obtain desired chimeric clones containing the G, NV, or G and NV gene substitutions (see Figure 1). DNA from all of the above-mentioned clones was sequenced by the dideoxy chain termination method to verify various gene substitution(s) in these clones.

**DNA transfection and recovery of chimeric viruses**: To generate recombinant parental and chimeric viruses, EPC cells were transfected with each of the eight different plasmid constructs (see Figure 1) along with the same supporting plasmids, pN, pP, and pL, of the MI03 or IHNV-220-90 strain, using the protocol described earlier [15,42]. After transfection, the cells were washed and maintained in Eagle’s MEM containing 10% FBS at 14 °C for 5 days. Cell monolayers were observed for the development of virus-induced CPE. After 5 days of incubation, the cells were submitted to three cycles of freeze–thawing. The supernatant was clarified by centrifugation at 8000× *g* in a microcentrifuge and used to inoculate fresh cell monolayers in T-25 flasks at 14 °C. The supernatant was harvested and clarified for the further characterization of the recombinant viruses.

**Preparation of virus stocks and plaque assay:** To prepare recombinant virus stocks, confluent EPC cells grown in T-75 flasks at 25 °C were infected at a multiplicity of infection (MOI) of 0.01 in MEM with 5% FBS. After 1 h of adsorption at 14 °C, the inoculum was removed, and the cells were incubated at 14 °C until extensive CPE was observed. The supernatant was collected 4–5 days post-infection, clarified, and stored at −80 °C. The titer of the virus was determined by plaque assay, as previously described [15]. The titer of the recombinant parental viruses (rVHSV-MI03 and rIHNV-220-90) and chimeric viruses ranged from 1.2 × 10^5^ to 1.1 × 10^7^ PFU/mL. Chimeric viruses based on the parental pVHSV-MI03 clone with substitution of G, NV, or G and NV genes from IHNV are referred to as the rVHSV-MI03 series, and chimeric viruses based on the parental pIHNV-220-90 clone with substitutions of G, NV, or G and NV genes from VHSV are referred to as the rIHNV-220-90 series (Figure 1).

**Characterization of the recombinant viruses rescued from cell culture:** To confirm the chimeric nature of the recombinant VHSVs and IHNVs, viral RNA was extracted from the supernatants of rVHSV- or rIHNV-infected cell cultures using an RNeasy Mini Kit (Qiagen, Germantown, MD, USA) as a template. Various primers synthesized in the initial cloning, sequencing, and mutagenesis of both genomes were used for RT-PCR amplification, as described previously [15,42]. First-strand synthesis was carried out in a tube containing 5 μL of RNA, which was denatured at 70 °C for 10 min in the presence of DMSO (3 μL), 1 μL forward gene-specific primer, 1 μL of 25 mM dNTPs, and snap-cooled on ice for 1 min. The reaction mixture containing 2 μL of 10× RT buffer, 2 μL of 0.1 M DTT, 4 μL of 25 mM MgCl_2_, 1 μL of Superscript III RT™, and 1 μL of RNase OUT™ was incubated at 50 °C for 1 h. PCR amplifications were carried out using a *pfx*50™ PCR kit (Invitrogen, Carlsbad, CA, USA), according to manufacturer’s instructions. Briefly, the following mixture was used for PCR amplification: 3 μL of cDNA, 2 μL of gene-specific forward and reverse primer mix; 5 μL of 10× PCR buffer [100 mM Tris–HCl (pH 9.0), 500 mM KC1, 1% Triton X-100], 2 μL of 25 mM MgCl_2_, 0.5 μL of *pfx*50 polymerase, and 37 μL of DEPC water, to make a final volume of 50 μL. The reaction was carried out in a thermal cycler (MJ Research Inc., Waltham, MA, USA), using the following program: denaturation at 94 °C for 30 s; annealing for 30 sec at 60 °C; and extension at 68 °C for 2 min. The PCR products were separated by agarose gel electrophoresis and were purified using a QIAquick gel extraction kit (Qiagen, Germantown, MD, USA). The obtained PCR products were directly submitted for DNA sequencing in the core facility of the Institute of Marine and Environmental Technology to confirm the presence of the VHSV or IHNV gene fragments and genetic markers introduced into the genome.

**Characterization of VHSV proteins synthesized in virus-infected cells by Western blot:** To detect the viral protein expression of two series of parental and chimeric viruses, EPC cells (in six-well plates) were infected with eight different viruses: four viruses of rVHSV-MI03 series and the other four of rIHNV-220-90 series. A time course study was carried out and the samples were harvested at 24 h, 48 h, 72 h, and 96 h post-infection. In total, 32 samples were collected. After lysis, the cellular proteins were resolved on four 12% sodium dodecyl sulfate–polyacrylamide gels (SDS-PAGE), transferred onto nitrocellulose membrane, and incubated with polyclonal rabbit anti-VHSV serum against MI03 strain [17]. VHSV proteins were detected with streptavidin–alkaline phosphatase and BCIP (5-bromo-4-chloro-3-indoyl phosphate p-toluidine salt)/NBT (p-nitro blue tetrazolium chloride) color development reagents.

**Viral growth curves in cell culture:** To analyze the in vitro growth characteristics of the rVHSVs and rIHNVs, confluent EPC cells were infected with the parental recombinant strain of rVHSV-MI03 and rIHNV-220-90 or chimeric recombinant rVHSV and rIHNV stocks at an MOI of 0.01 in individual T-25 flasks. Virus present in the infected cell culture supernatant was collected at different time intervals, clarified by centrifugation, and titrated on EPC cells by plaque assay, as described earlier [15].

**Virus challenge experiments in juvenile rainbow trout:** Two in vivo virus challenge experiments were conducted in compliance with guidelines provided by the Guide for the Care and Use of Laboratory Animals and the United States Public Health Service Policy on the Humane Care and Use of Laboratory Animals, under a protocol approved by the USGS Western Fisheries Research Center Institutional Animal Care and Use Committee. Research-grade naïve juvenile rainbow trout and yellow perch were provided by Clear Springs Foods Inc., and the WATER Institute at the University of Wisconsin, Milwaukee, respectively. The fish were reared in the Western Fisheries Research Center Aquatic Biosafety Level 2 (ABSL2) wet lab on flow-through, sand-filtered, UV-irradiated fresh lake water at 15 °C, and given pelleted feed (Skretting) at 1.5% body weight per day. Feed was withheld for 24 h prior to the start of each experiment, and during this time, the fish were gradually acclimated to 12 °C. The fish were then transferred to the Aquatic Biosafety Level 3 (ABSL3) laboratory of the Western Fisheries Research Center, where all in vivo experiments with recombinant viruses were conducted.

The first experiment tested the parental rIHNV and rVHSV viruses and the rIHNV-220-90-series chimeric viruses in both yellow perch and rainbow trout, with the wild-type IHNV strain 220-90 as a positive control treatment and mock-injected fish as a negative control treatment. Triplicate subgroups of 15 juvenile yellow perch (average weight 6.8 g) or rainbow trout (average weight 3.5 g) were challenged with the virus through the intraperitoneal injection of a dose of 1.0 × 10^5^ PFU per fish in 50 µL of phosphate-buffered saline (PBS), pH 7.4. Negative control groups were mock-infected by injection with PBS containing no virus. After injection, the individual replicate subgroups of fish were held in 30 L tanks with flow-through water at 12 °C, and fed three times per week. Two tanks of fish in each treatment group were monitored daily for morbidity and mortality for a period of 28 days. Fish that died during the challenge were collected daily and disease signs were recorded in the dead fish before storing them at −80 °C. A subset of fish that died in each treatment group (3 fish per treatment group unless a smaller total number of fish died) were titered for infectious virus by plaque assay [44] to confirm the presence of virus at titers that suggested viral infection as the likely cause of death. The third tank in each treatment was used to assess infection of fish with the recombinant viruses by collecting and euthanizing 5 fish per tank on days 3 and 7 after virus injection. These fish were stored at −80 °C and titered for infectious virus by plaque assay as above. The second experiment tested the parental rIHNV and rVHSV viruses and the rVHSV-MI03-series chimeric viruses in both juvenile yellow perch (average weight 2.7 g) and rainbow trout (average weight 1.0 g), with wild-type VHSV strain MI03 as a positive control treatment and mock-injected fish as a negative control treatment. The virus challenge and experimental procedures for the second experiment were the same as for the first experiment.

**Characterization of the chimeric viruses recovered from infected fish:** For each treatment group that had mortality, viral RNA from one fish that died late in the monitoring period was sequenced to verify that the recovered viruses contained the expected introduced substitutions of genes from each genotype of VHSV or IHNV. Genomic RNA was extracted from partially purified virus using an RNeasy Mini Kit (Qiagen) and were subjected to RT-PCR amplification to obtain cDNA fragments of the VHSV or IHNV genome, as described earlier. These DNA fragments were purified and sequenced to confirm the presence of introduced substitutions of G, NV, or G and NV protein genes into the VHSV or IHNV genome.

## 3. Results

**Chimeric cDNA clones and recombinant VHSV and IHNV:** The construction of infectious clones of the VHSV-MI03 and IHNV-220-90 strains has been described previously and the full-length clones were designated as pVHSV-MI03 and pIHNV-220-90, respectively [15,42]. These two parental clones were used to construct chimeric VHSV:IHNV clones. Figure 1 shows a schematic presentation of six full-length chimeric VHSV and IHNV cDNA clones constructed by exchanging the individual G, NV, or G and NV genes between the pVHSV-MI03 and pIHNV-220-90 clones. These plasmid constructs were used to recover a series of recombinant VHSVs and IHNVs (rVHSV and rIHNV). To verify the chimeric nature of each rVHSV and rIHNV, RNA was extracted from these viruses and used to obtain RT-PCR products. A sequence analysis of the RT-PCR products confirmed the chimeric nature of the rVHSVs and rIHNVs, as designed.

**Figure 2:**
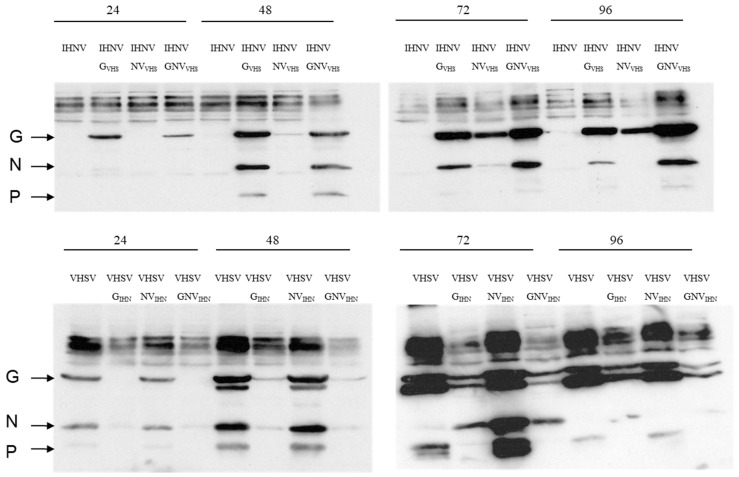
Western blot analysis of IHNV and VHSV proteins synthesized in virus-infected cells. EPC cells were mock-infected or infected with different rIHNV-220-90 series or rVHSV-MI03 series of chimeric viruses and harvested at 24, 48, 72, and 96 h post-infection. After lysis, cellular proteins were fractionated by 12% SDS-PAGE, blotted onto nitrocellulose membrane, reacted with polyclonal rabbit anti-VHSV serum (prepared against VHSV-MI03 strain), and detected with streptavidin–alkaline phosphatase and BCIP (5-bromo-4-chloro-3-indoyl phosphate p-toluidine salt)/NBT (p-nitro blue tetrazolium chloride) color development reagents. In the upper two quadrants, lanes 1–16 are cell lysates of the rIHNV-220-90 series of viruses containing the G, NV, or G and NV substitutions of VHSV-MI03, whereas in the lower two quadrants, lanes 1–16 are cell lysates of the rVHSV-MI03 series of viruses containing the G, NV, or G and NV substitutions of IHNV-220-90, as depicted. The arrows indicate the mobility of VHSV G, N, and P proteins, based on the reactivity with VHSV-specific polyclonal antibody.

**Analysis of recombinant viruses by immunostaining:** To detect the expression levels of the viral structural proteins synthesized in EPC cells infected with the rVHSV-MI03 series or rIHNV-220-90 series of chimeric viruses, the cellular proteins were separated by denaturing polyacrylamide gel electrophoresis, transferred onto nitrocellulose membrane, and probed with polyclonal antibody made against the MI03 strain. Figure 2 shows the bands produced by the reactivity of the VHSV structural G, N, and P proteins with VHSV polyclonal antibody after immunostaining. In the upper two quadrants, we detected the bands related to the cell lysates of the rIHNV-220-90 series of viruses containing the G, NV, or G and NV substitutions of VHSV-MI03. The reason for the low-intensity signal in some bands is because the antibody was made against the VHSV-MI03 strain, and it does not react strongly with the structural proteins of the IHNV strain. However, the results clearly show that the substitution of the G gene of rVHSV-MI03 into the rIHNV-220-90 series of chimeric viruses results in a robust signal for G and N proteins. In the lower two quadrants, we observe high-intensity signal in bands related to the cell lysates of the rVHSV-MI03 series of viruses because the VHSV antibody strongly reacted with the structural G, N, and P proteins of the VHSV strain, as depicted.

**Figure 3:**
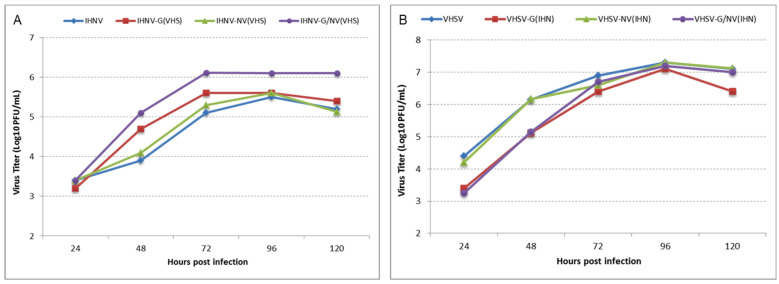
Replication kinetics of chimeric rIHNVs and rVHSVs in vitro. Monolayers of EPC cells were infected at an MOI of 0.01 with the chimeric viruses harboring substitutions of specific VHSV or IHNV gene(s) in the parental rIHNV-220-90 (panel **A**) or rVHSV-MI03 (panel **B**) viruses derived from respective pVHSV-MI03 or pIHNV-220-90 series of plasmids. The viruses were harvested at the indicated time points, and virus titers were determined by plaque assay. In the growth curve plots, the titers of recombinant chimeric viruses are color-coded: rIHNV and rVHSV (blue); rIHNV-G_VHS_ and rVHSV-G_IHN_ (red); rIHNV-NV_VHS_ and rVHSV-NV_IHN_ (green); and rIHNV-GNV_VHS_ and rVHSV-GNV_IHN_ (purple).

**Replication kinetics of chimeric viruses in vitro:** To analyze the in vitro growth characteristics of the chimeric rIHNVs and rVHSVs harboring reciprocal substitution(s) of the G, NV, or G and NV gene(s), a multiple-step growth curve study was carried out on EPC cells. Figure 3A shows the growth curves of the rIHNV-220-90 series, and Figure 3B shows the rVHSV-MI03 series at different time points between 24 and 120 h post-infection (p.i). All rIHNVs and rVHSVs were viable and replicated in vitro. In the rIHNV series (Figure 3A), all viruses grew to final titers very similar to the parental rIHNV (approximately 5 × 10^5^ PFU/mL) at 96 h p.i, except the rIHNV-GNV_VHS_*,* which had a ~0.5-log-higher titer than the rest. In the rVHSV series (Figure 3B), all viruses grew to final titers very similar to the parental rVHSV (approximately 1 × 10^7^ PFU/mL) at 96 h p.i, which is an almost 1.5-log-higher titer than the rIHNV series.

**Figure 4:**
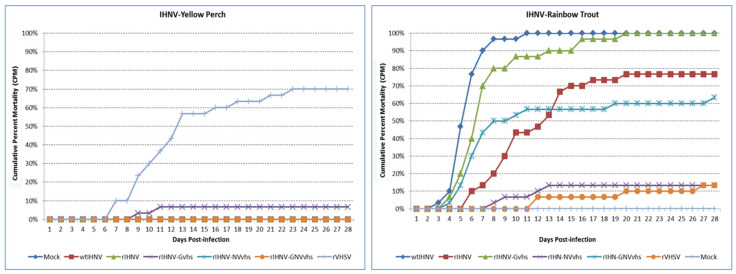
Fish mortality induced by the parental rIHNV-M and rVHSV-IVb, and the rIHNV-M series of chimeric viruses in juvenile yellow perch (**left panel**) and rainbow trout (**right panel**). Fish were challenged by intraperitoneal injection with 1 × 10^5^ PFU of wild-type IHNV, rIHNV, rIHNV-G_VHS_, rIHNV-NV_VHS_, rIHNV-GNV_VHS_, or rVHSV, or mock-infected with PBS. This experiment tested the impact of substitutions of the G, NV, or G and NV genes of VHSV-IVb into the rIHNV-M backbone clone. After challenge, the mortality of the fish was recorded daily. The average cumulative percent mortality (CPM) for duplicate subgroups of 15 fish is shown.

**Figure 5:**
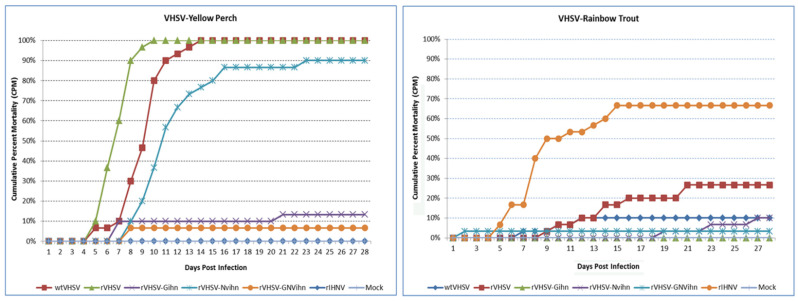
Fish mortality induced by the parental rVHSV-MI03 and rIHNV-M, and the rVHSV-IVb series of chimeric viruses in juvenile yellow perch (**left panel**) and rainbow trout (**right panel**). Fish were challenged by intraperitoneal injection with 1 × 10^5^ PFU of wild-type VHSV, rVHSV, rVHSV-G_IHN_, rVHSV-NV_IHN_, rVHSV-GNV_IHN_, or rIHNV, or mock-infected with PBS. This experiment tested the impact of substitutions of the G, NV, or G and NV genes of IHNV-M into the VHSV-IVb backbone clone. After challenge, the mortality of the fish was recorded daily. The average cumulative percent mortality (CPM) for duplicate subgroups of 15 fish is shown.

**Virulence of rIHNV- and rVHSV-series viruses in vivo:** Host-specific virulence was assessed as fish mortality after the intraperitoneal injection challenge of duplicate groups of 15 juvenile yellow perch or rainbow trout. Experiment 1 tested the rIHNV-220-90 series of chimeric rIHNV with relevant control groups (Figure 4), and experiment 2 tested the rVHSV-MI03 series of chimeric rVHSV with relevant control groups (Figure 5). In both experiments, the control wild-type virus strains and parental recombinant viruses demonstrated host-specific virulence, as expected. Table 1 shows that wt IHNV strain 220-90 and rIHNV-220-90 had high virulence in rainbow trout [70–100% final cumulative percent mortality (CPM)] and low virulence in yellow perch (0% CPM), while wt VHSV strain MI03 and rVHSV-MI03 had high virulence in yellow perch (70–100% CPM) and low virulence in rainbow trout (10–27%). There was no mortality in the mock treatment groups for either host species in either experiment.

In experiment 1, the three chimeric rIHNVs with G and/or NV genes from VHSV all exhibited low virulence in yellow perch (0–7%, Figure 4, left panel), so these gene substitutions did not alter the low perch virulence of the parental rIHNV-220-90. In rainbow trout (Figure 4, right panel), the chimeric rIHNV-G_VHS_ had high virulence (100% CPM), so the G gene from VHSV also did not alter the high trout virulence of the parental rIHNV-220-90. However, in rainbow trout, the chimeric rIHNV-NV_VHS_ had low virulence (13% CPM), while the rIHNV-GNV_VHS_ had moderately high virulence (63% CPM). This suggests that the VHSV NV gene alone causes a loss of trout virulence in rIHNV, but that the additional presence of the G gene of VHSV can compensate at least partially for that loss.

In experiment 2 (Figure 5, Table 1), the three chimeric rVHSVs with G and/or NV genes from IHNV all exhibited low virulence in rainbow trout (0–10% CPM), so these gene substitutions did not alter the low trout virulence of the parental rVHSV-MI03. In yellow perch, rVHSV-NV_IHN_ has high virulence (90% CPM), while rVHSV-G_IHN_ and rVHSV-GNV_IHN_ have low virulence (13 and 7% CPM). Therefore, for rVHSV, the NV gene of IHNV does not alter high perch virulence, but the G or G and NV genes of IHNV cause a loss of perch virulence.

Clinical signs consistent with IHN or VHS disease were observed in some fish that died in each experiment, with exophthalmia and external hemorrhages being the most common. Clinical signs occurred in the majority of yellow perch mortalities, including some from each parental and recombinant virus that caused mortality. In contrast, only approximately 20% of rainbow trout mortalities had visible signs. High titers of infectious virus (10^5^–10^7^ PFU/gram) were found in nearly all mortalities tested, including some from every treatment group in each fish host that had mortality. Finally, sequencing of the virus recovered from fish that died in each treatment group that had mortality confirmed the expected identity of the parental or chimeric virus.

**Infectivity of rIHNV- and rVHSV-series viruses in vivo:** In both in vivo experiments, fish were sampled at 3 and 7 days after virus challenge to assess the infection status of the fish in each treatment group. In experiment 1, infectious virus was recovered from several fish in treatment groups with each chimeric rIHNV in both hosts, including those that caused low or no final mortality. Average titers ranged from 4 × 10^2^ to 2 × 10^6^ PFU/g, but only fish in the rIHNV-220-90- and wt 220-90-positive control groups in rainbow trout had titers above the challenge inoculum of 1 × 10^5^ PFU per fish. In experiment 2, infectious virus was detected in several yellow perch challenged with each chimeric rVHSV virus, but rainbow trout inoculated with the same chimeric rVHSV did not have detectable virus. Where virus was detected, the average titers ranged from 3 × 10^4^ to 8 × 10^6^ PFU/g, but only fish in two groups, rVHSV-MI03 and rVHSV-NV_IHN_ in yellow perch, had average titers above 1 × 10^5^ PFU/g. These results suggest that the rIHNV and rVHSV tested here can at least persist transiently for up to 7 days in yellow perch, and the rIHNV can persist for 7 days in rainbow trout. However, since most treatment groups did not have virus titers above the quantity injected as inocula, we cannot be certain whether the virus recovered from the sampled fish represents virus replication in vivo or persistence of the virus from the inocula.

## 4. Discussion

The work presented here was designed to test whether the G and NV genes of an IHNV-M strain and a VHSv-IVb strain are responsible for the host-specific virulence of these important fish rhabdoviruses. The parental virus strains have reciprocal virulence phenotypes in which the IHNV 220-90 (M genogroup) is highly virulent in rainbow trout but not yellow perch, and VHSV MI03 (genotype IVb) is highly virulent in yellow perch but not rainbow trout. To evaluate the virulence of IHNV-220-90 and VHSV-MI03 chimeras in rainbow trout and yellow perch, we employed the existing IHNV-220-90 and VHSV-MI03 infectious clones [15,42] and generated six chimeric viruses by exchanging the G, NV, or G and NV genes into each parental clone (Figure 1). An evaluation of their replication kinetics in vitro confirmed that all chimeric viruses were viable and replicated in the fish cell line EPC. This provides additional examples of the previously reported flexibility of rhabdovirus genomes in being able to accommodate exchanges of genes from many heterologous species [45,46,47,48,49,50,51,52,53]. In the rIHNV series, all viruses grew to final titers very similar to the parental rIHNV (approximately 5.5 log_10_ PFU/mL), except the rIHNV-GNV_VHS_, which had a titer about 0.5-log higher (Figure 3A). In the rVHSV series, all viruses grew to final titers similar to the parental rVHSV (approximately 7 log_10_ PFU/mL), which was 1.5-log higher than the titers for the rIHNV series (Figure 3B).

In the in vivo testing of the rIHNV- and rVHSV-series viruses, all positive control groups performed as expected, with wt IHNV strain 220-90 and rIHNV-220-90 having high virulence in rainbow trout and low virulence in yellow perch, while wt VHSV strain MI03 and rVHSV-MI03 had high virulence in yellow perch and low virulence in rainbow trout. The results for the chimeric viruses in each series fell into two general virulence categories similar to the high and low virulence levels observed in the control groups. Thus, the final average CPM values for all treatment groups in both experiments fell between 0 and 27%, designated as low virulence, or between 63 and 100%, designated as high virulence. A summary of the virulence phenotypes defined for each recombinant virus in the rIHNV and rVHSV series is shown in Table 2.

For the interpretation of the results of reverse genetics studies such as this one, the most conclusive outcomes for identifying virulence determinants would be the observation of increased virulence for chimeric viruses in the host species where the parental recombinant virus has low virulence. In this host, if the substitution of the G and/or NV genes from the heterologous virus resulted in an increase in high virulence, this would mean that those genes carry the determinants for high virulence in that host. In our results, there were no cases where gene exchanges caused an increase from low to high virulence relative to the parental clone. As summarized in Table 2, all rIHNV-series viruses tested in yellow perch had low virulence, and all rVHSV-series viruses tested in rainbow trout had low virulence. This indicates that the determinants for the high virulence of IHNV-M in rainbow trout and for the high virulence of VHSV-IVb in yellow perch do not reside in the G and NV genes of those viruses, but must be in other gene(s), or in a combination of genes in addition to the G and NV. This observation is consistent with previous results from other investigators and ourselves, who used various molecular constructs of fish rhabdoviruses and showed that the G and NV genes do not contain the primary determinants for host-specific virulence [45,48,49,51]. This does not preclude the possibility that G and NV genes contribute to virulence in some way, but they do not define the host-specific nature of virulence for these viruses in these hosts.

In general, observations of reduced virulence due to gene exchanges are more difficult to interpret in reverse genetics studies because reduced virulence may be caused by either a loss of virulence determinants or impacts on general viral fitness, which might be due to incompatibility between viral proteins from heterologous viruses. In our results, there were three cases where gene exchanges resulted in a reduction in virulence relative to the high virulence of the parental clone (Table 2, yellow highlights). In the rIHNV series tested in rainbow trout, the substitution of the G gene from VHSV did not alter the high virulence phenotype of the parental rIHNV. However, the substitution of the NV gene of VHSV did reduce the virulence to low. Although this might suggest that the IHNV NV gene is essential for the high virulence of IHNV in rainbow trout, the double substitution of both G and NV genes from VHSV returns the virulence level to high, albeit at the low end of the high virulence range. The fact that the rIHNV-GNV_VHS_ chimera causes 63% mortality in rainbow trout means that the NV of IHNV is not essential for trout virulence. It appears instead that the NV of VHSV can support trout virulence if the homologous G gene from VHSV is also present in order to function efficiently. This strongly suggests that interaction between the NV and G proteins of VHSV is important for their function, which can then support viral virulence or fitness in the rIHNV background. It is interesting to note that the rIHNV-GNV_VHS_ chimeric virus is the one that replicated to higher titer than the parental rIHNV or other rIHNV chimeric viruses in vitro (Figure 3), possibly suggesting that the G and NV from VHSV are interacting to confer the higher replication phenotype of rVHSV in vitro. In general, the findings from our rIHNV-series viruses in rainbow trout are in agreement with the earlier report by Einer-Jensen et al. [45] in which the substitution of NV_VHS_ significantly reduced the virulence of chimeric rIHNV in rainbow trout, whereas G_VHS_ substitution increased the virulence and a G + NV chimeric virus was not tested.

For the rVHSV-series viruses in yellow perch, two of the three chimeric viruses caused a reduction to low virulence relative to the high virulence of the parental rVHSV (Table 2, yellow highlights). The two chimeras with either G or G and NV from IHNV had low virulence, while the chimeric viruses with the NV substitution remained high virulence in yellow perch. This suggests that the G gene of IHNV is responsible for the loss of perch virulence in the rVHSV context, and that, in this case, the loss of virulence is not compensated for by the presence of the homologous NV gene from IHNV.

As shown in Table 2, non-reciprocal outcomes for exchanges of genes between IHNV and VHSV are evident in the loss-of-virulence patterns, i.e., in the results from the rIHNV-series viruses tested in rainbow trout or the rVHSV-series viruses tested in yellow perch. For the NV gene, the exchange of the VHSV NV gene into rIHNV (rIHNV-NV_VHS_) resulted in a loss of trout virulence, while the substitution of the IHNV NV gene into rVHSV (rVHSV-NV_IHN_) did not alter the high perch virulence of rVHSV. Similarly, for the G and G + NV genes, exchanges from VHSV into rIHNV (rIHNV-G_VHS_ and rIHNV-GNV_VHS_) did not alter the high trout virulence of rIHNV, while exchanges of genes from IHNV into rVHSV (rVHSV-G_IHN_ and rVHSV-GNV_IHN_) did cause a loss of perch virulence. At present, it is difficult to understand what these observations mean, but they suggest that the interactions of the G and NV proteins, possibly with host components, are different for IHNV and VHSV. This may somehow be related to the narrow and broad host ranges of IHNV and VHSV, respectively, but links between these observations and virus–host-specific mechanisms will require a better understanding of novirhabdovirus protein functions and interactions in the future.

In previous studies, Biacchesi et al. [46] was first to develop an infectious clone of a French IHNV strain that has high virulence in rainbow trout [13,46]. Using this IHNV clone, inter-species chimeras were created in which the G, NV, and M genes were independently substituted with the corresponding gene from a European strain of VHSV that also had high virulence in rainbow trout (genotype Ia). In each case, these chimeras were viable in vitro in fish cell lines, and they exhibited high virulence in vivo for rainbow trout [13,47,48]. Using the same infectious clone, other chimeras were produced containing the G genes from spring viremia of carp virus (SVCV, fish rhabdovirus), vesicular stomatitis virus (VSV, mammalian rhabdovirus), or various strains of VHSV or IHNV. In each case, viable chimeric recombinant viruses were recovered and shown to replicate in fish cell lines [45,49,50]. For those tested in vivo (all but the rIHNV-Gvsv), these chimeric rIHNVs had high virulence in rainbow trout [48,49,50]. In many of these studies, the question of host-specific virulence was not addressed because the parental viruses that contributed to the chimeras did not differ in phenotype and both had high virulence in rainbow trout. In our previous work, we addressed the host-specific virulence within strains of VHSV by constructing an infectious clone of a trout-virulent VHSV strain (DK-3592B, genotype Ia, Denmark) and systematically exchanging the six viral genes, either individually or as selected gene combinations, between this clone and the trout-avirulent VHSV clone (VHSV strain MI03, genotype IVb). Testing of 16 chimeric rVHSV viruses in rainbow trout showed that the M, G, NV, and L genes did not contain trout virulence determinants, but the N and P proteins are major determinants of virulence of VHSV in rainbow trout [51,52].

## 5. Conclusions

To investigate the host-specific virulence of IHNV and VHSV, we used existing IHNV and VHSV infectious clones and generated chimeric viruses by exchanging the G, NV, or G and NV genes into each parental clone. These chimeras were used to challenge the groups of rainbow trout and yellow perch to assess the virulence capacity. Our results do not show any cases of gain of virulence, confirming previous results that the G and NV genes do not carry determinants of host-specific virulence for IHNV or VHSV. It is clear from our work and the work of others that the virulence determinants of fish rhabdoviruses are more complex than many other viruses, and not based on a simple viral receptor mechanism driven by the viral glycoprotein. Our results do show some G and/or NV gene substitutions that cause the loss of high virulence. These are not conclusive regarding virulence determinants, but can be informative about interactions between viral proteins. In this work and in our previous studies, recombinant viruses were delivered to juvenile fish by intraperitoneal injection [51,52]. Virulence phenotypes may differ if viruses are delivered by the natural route of immersion, as described for other recombinant VHSVs [53]. The results presented here do not provide final answers to the question of which viral genes are responsible for the trout virulence of IHNV-M or yellow perch virulence of VHSV-IVb, but they do contribute intriguing pieces to the puzzle of host-specific virulence. This work, combined with the published and future work of others, will lead to a better understanding of how virulence is determined and evolves in fish rhabdoviruses, through host jumps and adaptation to novel host species.

## Figures and Tables

**Table 1:** Mortality in in vivo injection challenge studies in juvenile yellow perch and rainbow trout.

	Virus Treatment	Yellow Perch Final Ave. CPM ^a^ (Individual Tank Mortality)	Rainbow Trout Final Ave. CPM ^a^ (Individual Tank Mortality)
(a) Experiment 1, rIHNV-220-90 series			
	wt IHNV 220-90	0% (0/14, 0/14)	**100%** (15/15, 15/15)
	rIHNV-220-90	0% (0/15, 0/15)	**77%** (14/15, 9/15)
	rIHNV-G_VHS_	7% (2/15, 0/14)	**100%** (15/15, 15/15)
	rIHNV-NV_VHS_	0% (0/15, 0/15)	13% (1/15, 3/15)
	rIHNV-GNV_VHS_	0 % (0/15, 0/15)	**63%** (7/15, 12/15)
	rVHSV-MI03	**70%** (9/15, 12/15)	13% (3/15, 1/15)
	Mock, no virus	0% (0/15, 0/15)	0% (0/15, 0/15)
(b) Experiment 2, rVHSV-MI03 series			
	wt VHSV MI03	**100%** (15/15, 15/15)	10% (3/15, 0/15)
	rVHSV-MI03	**100%** (15/15, 15/15)	27% (5/15, 3/15)
	rVHSV-G_IHN_	13% (2/15, 2/15)	0% (0/15, 0/15)
	rVHSV-NV_IHN_	**90%** (14/15, 13/15)	10% (2/15, 1/15)
	rVHSV-GNV_IHN_	7% (1/15, 1/15)	3% (0/15, 1/15)
	rIHNV-220-90	0% (0/15, 0/15)	**70%** (10/15, 11/15)
	Mock, no virus	0% (0/15, 0/15)	0% (0/15, 0/15)

^a^ CPM is shown as the average final cumulative percent mortality observed in duplicate tanks of 14–15 fish, with CPM levels considered high (ranging 63–100%) in bold type and low (ranging 0–27%) in plain type.

**Table 2:** Host-specific virulence phenotypes of recombinant rIHNV-series and rVHSV-series viruses in juvenile rainbow trout and yellow perch.

Recombinant Virus	Source of N, P, M, L	Source of G	Source of NV	Yellow Perch Virulence (CPM)	Rainbow Trout Virulence (CPM)
rIHNV-220-90	I	I	I	LOW (0)	HIGH (77)
rIHNV-G_VHS_	I	V	I	LOW (7)	HIGH (100)
rIHNV-NV_VHS_	I	I	V	LOW (0)	LOW (13)
rIHNV-GNV_VHS_	I	V	V	LOW (0)	HIGH (63)
					
rVHSV-MI03	V	V	V	HIGH (100)	LOW (27)
rVHSV-G_IHN_	V	I	V	LOW (13)	LOW (0)
rVHSV-NV_IHN_	V	V	I	HIGH (90)	LOW (10)
rVHSV-GNV_IHN_	V	I	I	LOW (7)	LOW (3)

Green boxes show coding regions of recombinant viruses derived from IHNV-220-90, and purple boxes are coding regions derived from VHSV-MI03. Host-specific virulence phenotypes of recombinant viruses from in vivo challenge studies are shown on the right (Figure 4 and Figure 5). Virulence phenotypes are shown as high (final average cumulative percent mortality 63–100%) or low (0–27%) (see Table 1). Host-specific virulence of parental rIHNV and rVHSV phenotypes are shown in red texts. The text highlighted in yellow boxes are the only three cases where gene exchanges altered the host-specific virulence phenotype of either parental clone.

## Data Availability

Data are not currently available from the funding organization, the University of Maryland. Please contact the organization for further information.
